# A composite structure based on reduced graphene oxide and metal oxide nanomaterials for chemical sensors

**DOI:** 10.3762/bjnano.7.133

**Published:** 2016-10-10

**Authors:** Vardan Galstyan, Elisabetta Comini, Iskandar Kholmanov, Andrea Ponzoni, Veronica Sberveglieri, Nicola Poli, Guido Faglia, Giorgio Sberveglieri

**Affiliations:** 1 Sensor Lab, CNR, National Institute of Optics (INO), Via Valotti 9, 25133 Brescia, Italy; 2 Sensor Lab, Department of Information Engineering, University of Brescia, Via Valotti 9, 25133 Brescia, Italy; 3 Department of Mechanical Engineering, The University of Texas at Austin, Austin, TX 78712, USA

**Keywords:** chemical sensors, reduced graphene oxide (RGO), volatile organic compounds, zinc oxide (ZnO)

## Abstract

A hybrid nanostructure based on reduced graphene oxide and ZnO has been obtained for the detection of volatile organic compounds. The sensing properties of the hybrid structure have been studied for different concentrations of ethanol and acetone. The response of the hybrid material is significantly higher compared to pristine ZnO nanostructures. The obtained results have shown that the nanohybrid is a promising structure for the monitoring of environmental pollutants and for the application of breath tests in assessment of exposure to volatile organic compounds.

## Introduction

Hazard analysis of critical control point (HACCP) systems address food safety through the identification and control of the major food risks, i.e., biological, chemical and physical hazards. The metabolic activity of microorganisms in dairy foods leads to breakdown of chemical compounds into alcohol and organic acids [1]. Consequently, early detection of ethanol on the surface of food products is necessary in order to avoid the subsequent hazards and to take steps to decrease the spoilage rate in food products. Besides, ethanol and acetone can be assigned to specific pathologies and may be utilized as breath markers [2]. In particular, acetone is a selective breath marker and the presence of its certain concentrations in breath can reflect metabolic products of diabetes [3]. Due to the development of chemical industries acetone is one of the most commonly used volatile organic compounds (VOCs) and can cause dangerous health issues such as blindness, allergies and unconsciousness [4]. Therefore, the detection of VOCs such as acetone and ethanol is essential.

Nowadays, chemical and physical methods for environmental and medical diagnostics are rapidly developing. Medical monitoring technologies mainly focus on breath and blood for clinical diagnostics [5–6]. During the last decades, different types of sensors were fabricated for environmental and health monitoring. Among the different detection systems, chemical sensors based on metal oxide nanomaterials are highly demanded because of their high sensitivity, small size, low cost and low power consumption [7–8]. Metal oxide sensors can detect ethanol and acetone only at high operating temperatures (≥300 °C) [7,9–10]. ZnO is a extensively studied and inspiring material due to its unique properties, namely the wide bandgap and large exciton binding energy [11]. Most of the literature is focused on the synthesis of ZnO films, nanowires and ZnO-based hybrids for applications in opto-electronics as well as in gas sensors [7,12–15]. ZnO has several advantages regarding the application in sensor structures. However, there are many obstacles (high resistivity and operating temperature, sensitivity and selectivity) with respect to the application of ZnO nanomaterials in chemical gas sensors that need to be overcome [7,14].

Hybrid structures composed of two or more different materials with diverse functional properties are of great interest to develop advanced composite materials for numerous applications [16–17]. Graphene-based materials are very attractive because of their specific properties and large surface area [18–19]. Several new kinds of graphene-based structures were developed in rapid succession, which raises great interest nowadays and the exclusive properties of these materials make them a suitable candidate for various applications [20–21]. Recently we have shown that the functionalization of ZnO with reduced graphene oxide (RGO) sheets improved its sensing performance for NO_2_ and H_2_ [22]. Abideen et al. also improved the response of ZnO towards H_2_ preparing ZnO nanofibers loaded with reduced graphene oxide [23]. These recent studies indicate that the combination of graphene and its modified structures with ZnO nanomaterials may open new perspectives for the fabrication of ZnO-based chemical sensors.

In this paper, we describe a hybrid nanomaterial consisting of RGO and ZnO with a highly improved performance in sensing the VOCs ethanol and acetone. The highly improved sensing behavior of the obtained structures shows that our hybrid nanomaterial may be used to fabricate gas sensor devices for the detection of VOCs.

## Experimental

The method used for fabricating the ZnO nanostructures is similar to that described in our previous work [24]. Thin films of metallic Zn with a thickness of 600 nm were deposited on 2 mm square alumina substrates by means of radio frequency (RF) magnetron sputtering. Thin deposited films of Zn were anodized in 2 M oxalic acid dihydrate (C_2_H_2_O_4_·2H_2_O) containing ethanol using a two-electrode system. A platinum foil was used as a counter electrode and the anodization process was carried out at room temperature. The obtained structures were zinc oxalate dihydrate (ZnC_2_O_4_·2H_2_O). The as-prepared samples were transformed to crystalline ZnO by thermal annealing in a furnace at 400 °C as we have described in [25].

We prepared the composite material using the method described in [22]. We produced graphite oxide from natural graphite (SP-1, Bay Carbon) by means of modified Hummers method [26]. Then, we prepared aqueous dispersions of GO by stirring graphite oxide solids in pure water (18.0 MΩ·cm resistivity, purchased from Barnstead) for 3 h and sonicated the resulting mixture (VWR B2500A-MT bath sonicator) for 45 min. This method yields well-dispersed GO in water. We drop-cast the obtained aqueous dispersion of GO onto ZnO nanostructures and annealed the prepared hybrid material in a furnace at 250 °C in an atmosphere of 20% O_2_ and 80% Ar for 1 h.

The surface morphology of the samples was studied by means of a LEO 1525 scanning electron microscope (SEM) equipped with a field emission gun. Energy dispersive X-ray spectroscopy (EDX) was used to quantify the elemental composition of the obtained materials. GO platelets were deposited onto Si substrates and characterized by Raman spectroscopy (WITec Micro-Raman Spectrometer Alpha 300, λ = 532 nm, 100× objective).

To perform gas sensing measurements, the platinum electrodes and the heater were deposited on the front and rear sides of the alumina substrate, respectively. During gas sensing tests, the conductance of the samples was monitored by means of the volt-amperometric technique and the applied voltage during the measurements was 1 V. We recorded the resistance of the structures every 30 s. Measurements were carried out by means of a flow-through technique at atmospheric pressure, using a constant synthetic airflow (0.3 L/min) as carrier gas for the analyte dispersion. During the experiments the relative humidity was 50%. Gas response (R) was defined as [*R* = (*G*_f_ − *G*_0_)/*G*_0_], where *G*_0_ is the sample conductance in air, and *G*_f_ is the sample conductance in presence of the analyte gas.

## Results and Discussion

### Morphological and structural characteristics

**Figure 1:**
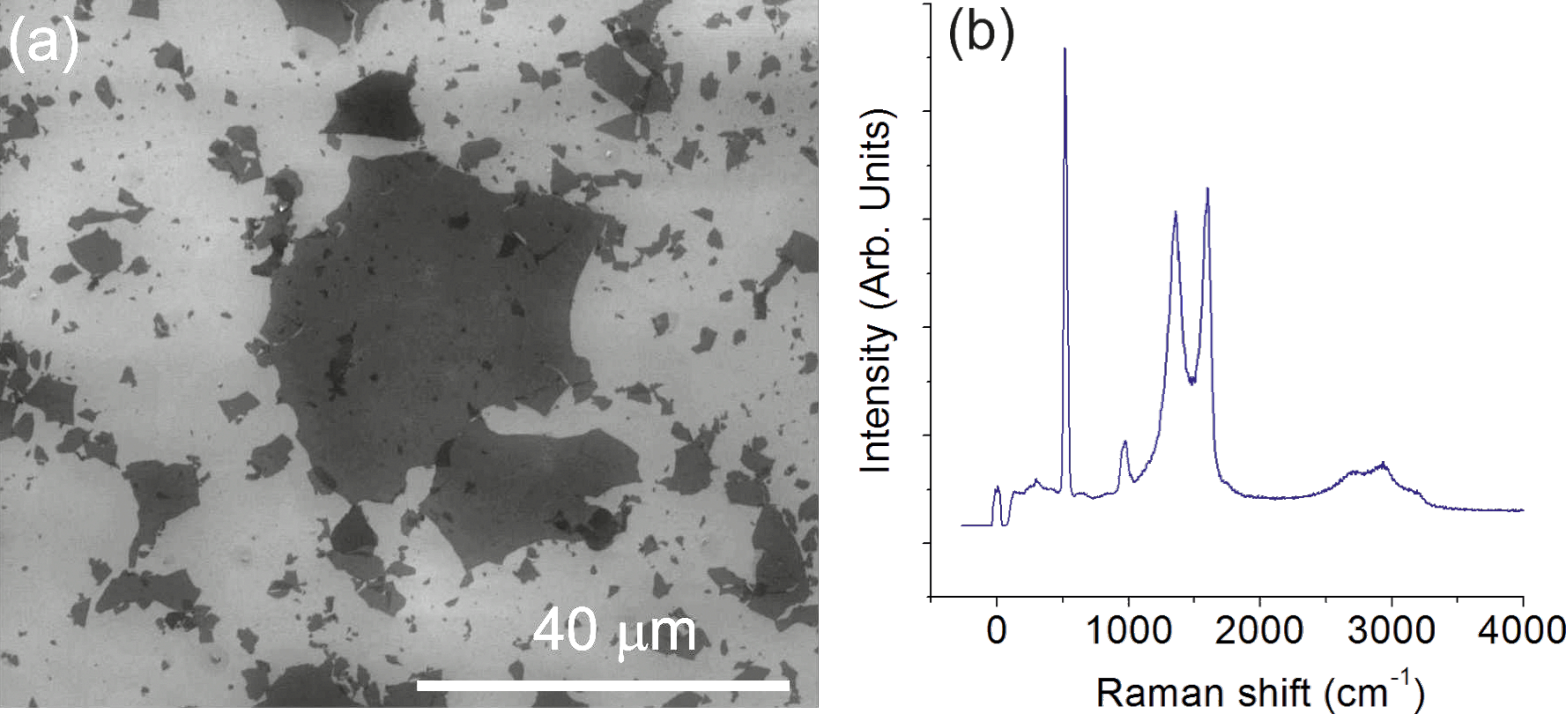
SEM image (a) and Raman spectrum (b) of the GO platelets deposited on SiO_2_/Si wafer.

For characterizing the GO samples with SEM, an aqueous dispersion was spin-coated onto a silicon wafer at 4000 rpm for 2 min. A typical SEM image of the GO platelets is shown in Figure 1a. The lateral size of GO platelets exhibits a wide distribution ranging from several nanometers up to about 20 micrometers. Figure 1b shows the typical Raman spectrum of GO platelets with high intensity D (≈1350 cm^−1^) and G (≈1580 cm^−1^) peaks. The Raman D band of graphene is activated by the defects that cause an inter-valley double resonance involving transitions near two inequivalent K points at neighboring corners of the first Brillouin zone of graphene [27]. Due to the decrease in size of the in-plane sp^2^-hybridzed domains after extensive oxidation and ultrasonic exfoliation, the GO exhibits a broad and intense D band in its Raman spectrum [28]. The intensity ratio between D and G peaks (*I*_D_/*I*_G_ = 0.94) also indicates the high defect concentration in GO platelets. High intensity peaks at about 520 cm^−1^ and 950 cm^−1^ in the Raman spectrum can be attributed to the silicon substrate.

**Figure 2:**
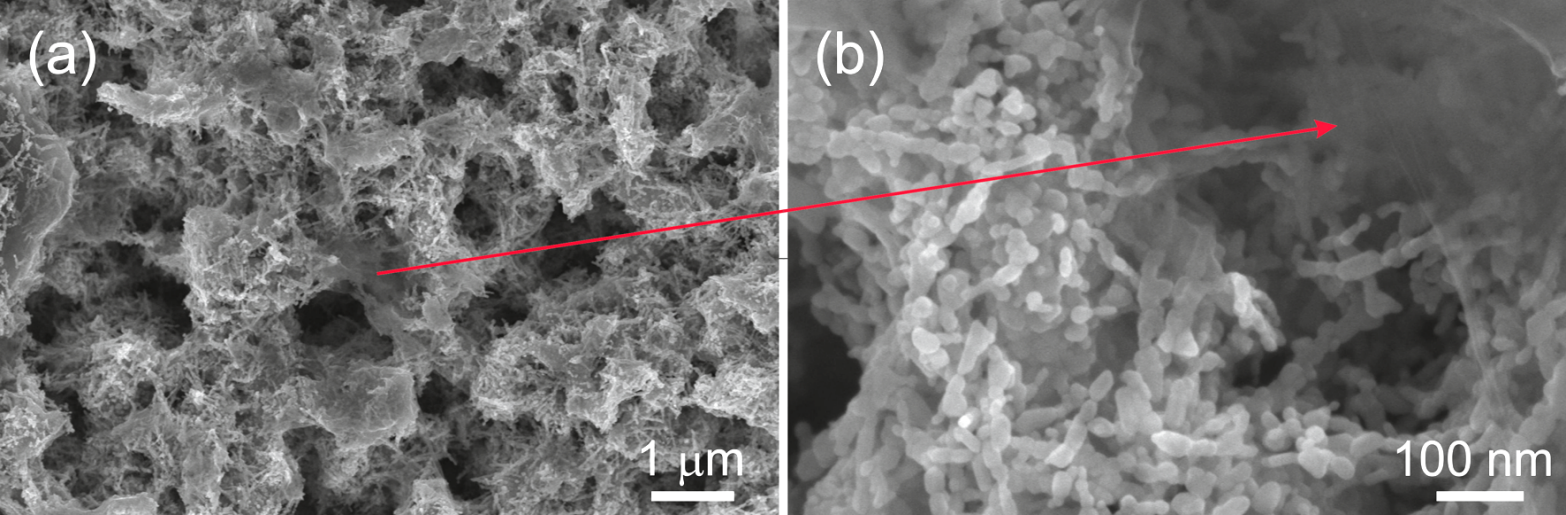
SEM images of the obtained samples based on graphene and zinc oxide at low (a) and high (b) magnification.

The morphology of the obtained hybrid material at different magnifications is shown in Figure 2. The ZnO nanoparticles have an average diameter of ca. 20 nm and form a porous structure of chain-like agglomerates [24]. As can be seen in the images GO platelets decorate the ZnO nanoparticles.

**Figure 3:**
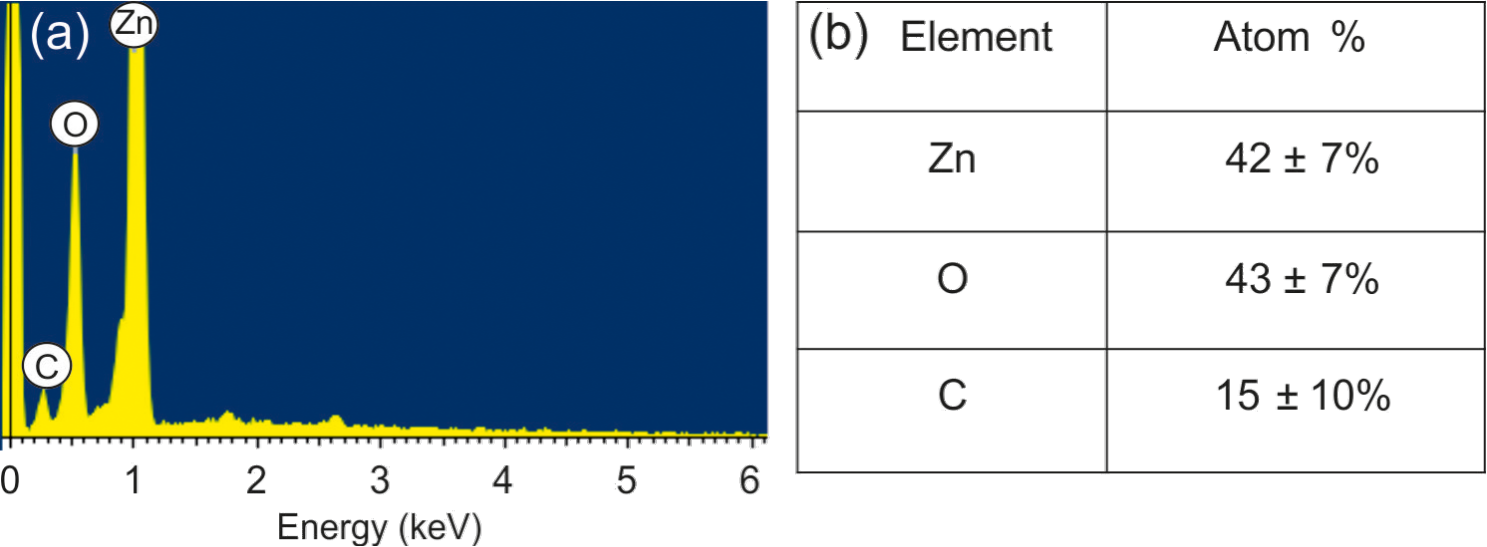
(a) EDX spectrum and (b) quantitative analysis of the hybrid structure based on GO and ZnO annealed at 250 °C.

Figure 3 reports the EDX spectrum and the quantitative analysis of the prepared structure. The morphological and the compositional studies confirm that the surface of ZnO nanomaterial is partially covered by GO. The variation of the C/O ratio in the GO platelets was checked by EDX before and after the thermal treatment at 250 °C. The EDX observations indicate that the C/O ratio increased due to the treatment, which means that GO was partially reduced (Table 1). The obtained results are in agreement with the our previous work on a similar material for the detection of explosive and toxic gases [22].

**Table 1:** The results of the compositional analysis of as-prepared and annealed samples (at 100 and 250 °C) on SiO_2_/Si wafers.

annealing temperature (°C)	C (atom %, ±3%)	O (atom %, ±10%)

as-prepared sample	62	38
100	69	31
250	77	23

### VOC sensing performance

The sensing measurements were performed with ethanol and acetone at working temperatures ranging from 20 to 250 °C. Before each measurement, we stabilized the obtained structures for 8 h at the operating temperature in ambient air. The sensing tests revealed that both pure ZnO and the hybrid materials exhibit enhanced response kinetics and response amplitudes when the operating temperature is increased. As a result, the best sensing results were obtained at the maximum sensor temperature (250 °C).

**Figure 4:**
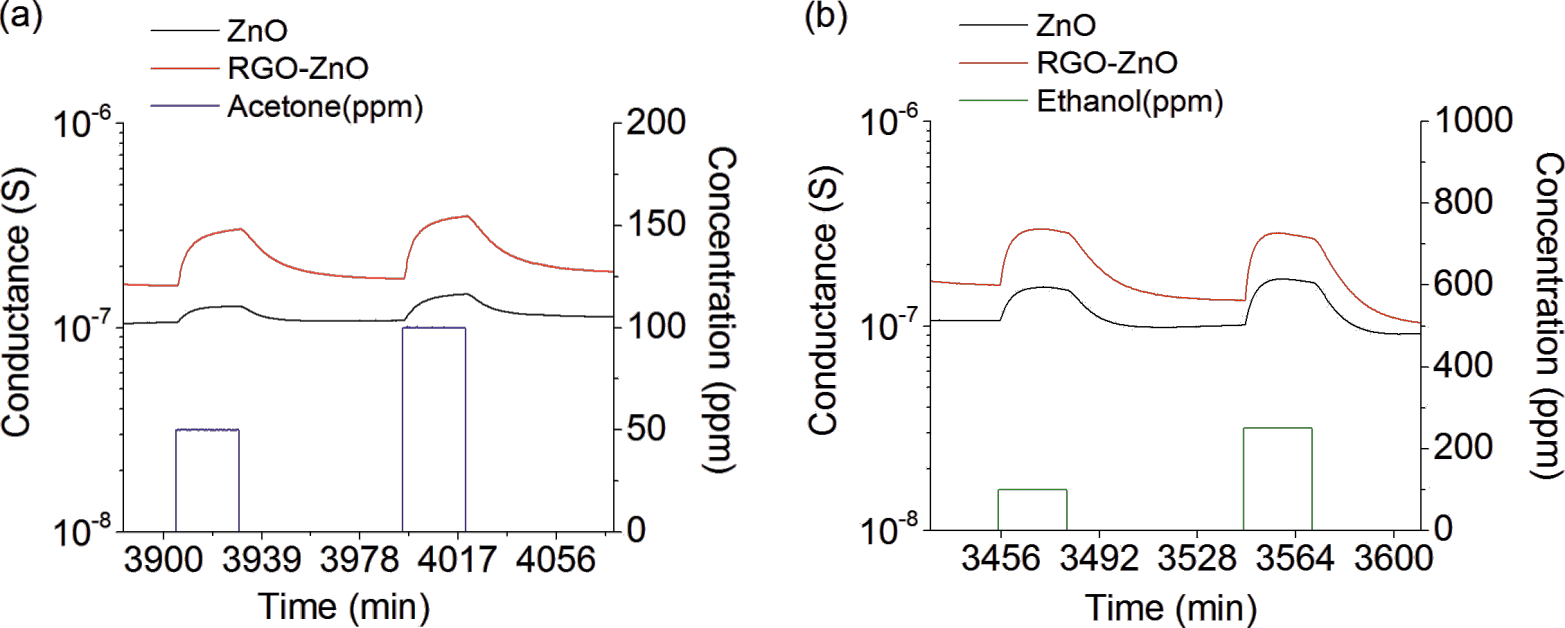
Dynamical response of ZnO and RGO–ZnO structures at 250 °C and RH = 50% @ 20 °C: (a) towards 50 and 100 ppm of acetone and (b) towards 100 and 250 ppm of ethanol.

**Figure 5:**
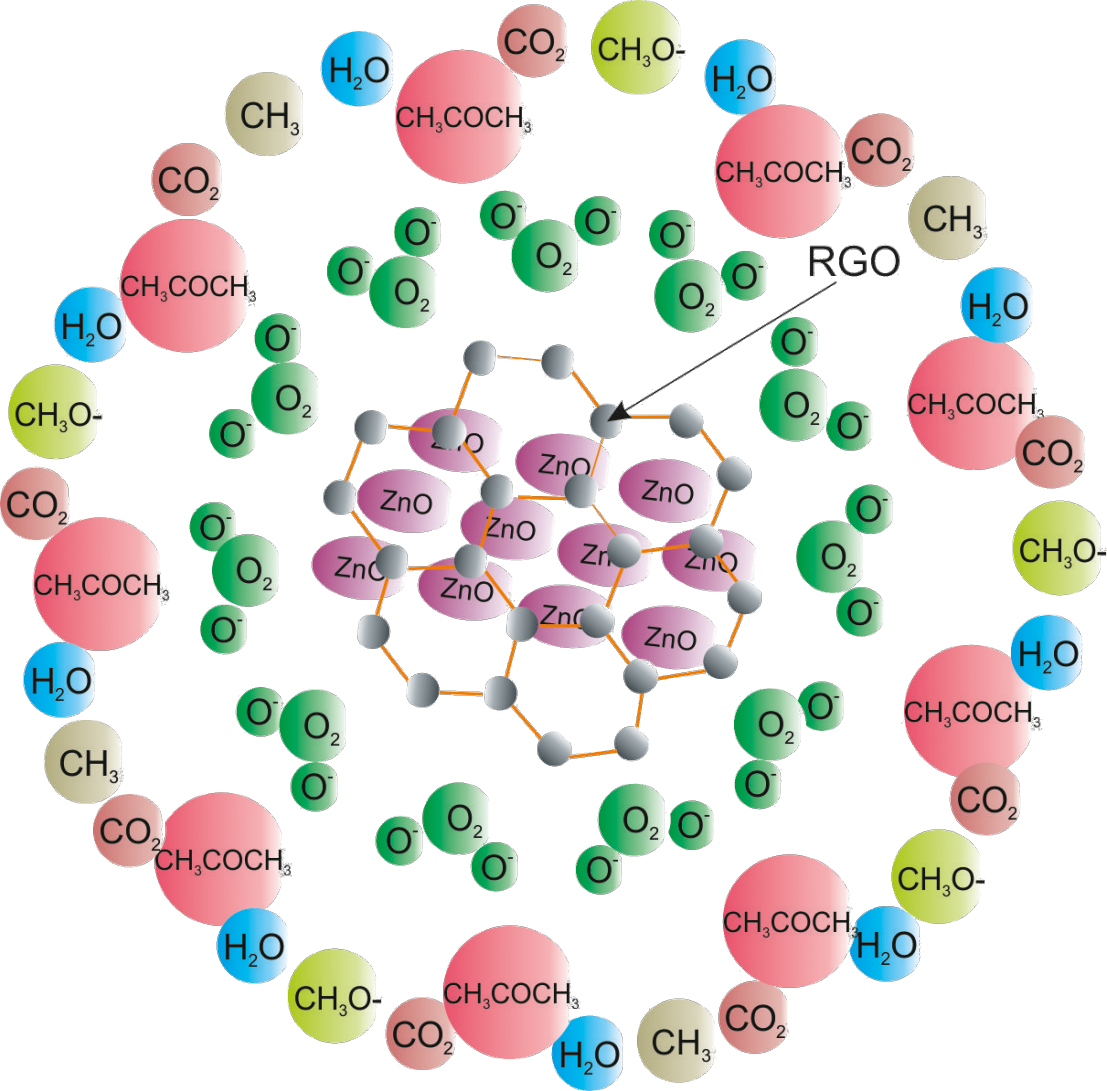
Schematic diagram of the sensing mechanism between acetone and the RGO–ZnO structure: Oxygen is absorbed on the structure creating O^−^ species (Equation 1). Upon exposure to acetone, acetone molecules adsorb and donate the electrons to the adsorbed oxygen species (Equations 2–4) forming CO_2_, H_2_O and other compounds such as CH_3_, CH_3_O^−^.

Figure 4 shows the response and the recovery curves of the prepared ZnO and RGO–ZnO structures towards acetone and ethanol at an operating temperature of 250 °C. Since ZnO is an n-type semiconductor, when it is exposed to air at elevated temperatures the oxygen molecules are adsorbed on the surface of the material generating the electron depletion layer. The adsorbed oxygen mainly forms O^−^ ions on the material surface (Equation 1) at temperatures of 200 °C or above [29]. As the reducing gas such as acetone (or ethanol) was introduced to the test chamber, the gas reacts with the adsorbed O^−^ ions. This results in less ionic oxygen species on the surface and, consequently, in an increased conductance of the structures. The proposed reactions with acetone and ethanol that lead to a sensing signal are resumed in Equations 2–4 [30–31] and Equation 2 [32], respectively. A schematic representation of the sensing mechanism between the acetone and the RGO–ZnO structure is shown in Figure 5. As can be seen in Figure 4, an obvious increase of conductance was observed by exposing the sensor to acetone and ethanol indicating that the nanohybrid structure is able the detect VOCs. The conductance of the sensor after the gas test was recovered to the initial value proving a reversible interaction between the analyte gases and the structure.

[1]
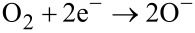


[3]
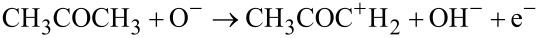


[4]
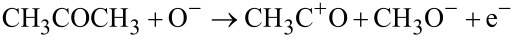


[5]
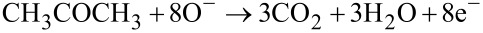


[2]
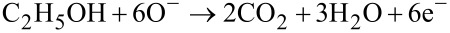


**Figure 6:**
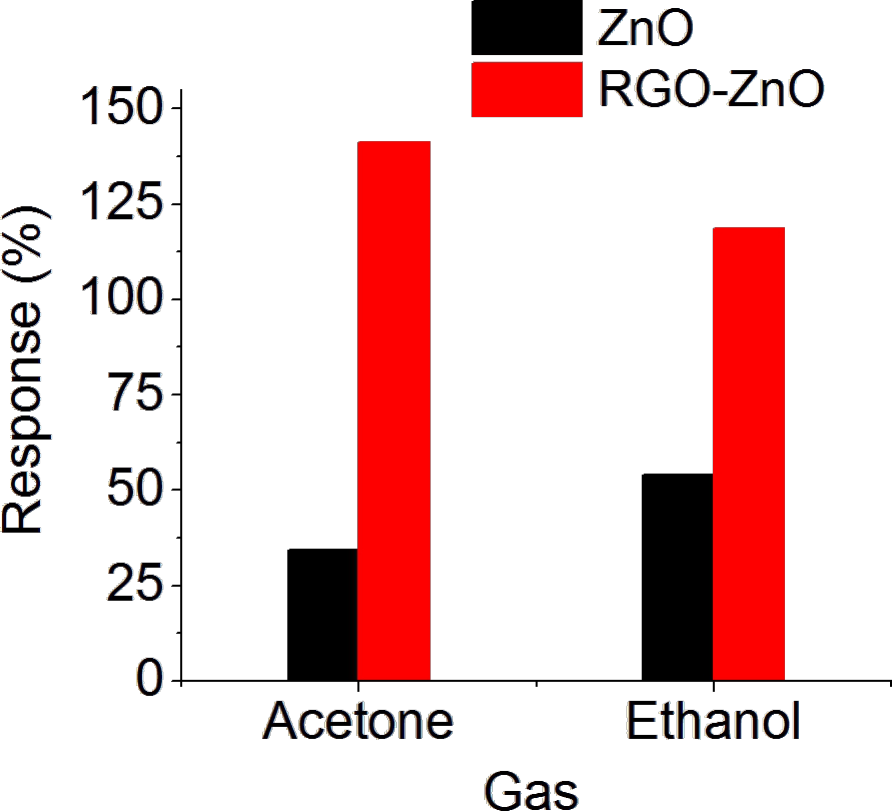
Response of RGO–ZnO and pristine ZnO nanostructures towards 100 ppm acetone and ethanol at a working temperature of 250 °C and in humid air (relative humidity RH = 50% @ 20 °C).

We compared the sensing performance of the RGO–ZnO hybrid structure with a pristine ZnO nanostructure (Figure 6). The response of the nanohybrid towards both gases is much higher compared to the pristine ZnO. The response values of RGO–ZnO and ZnO towards 100 ppm of acetone are 140 and 35%, respectively. The response values towards 100 ppm of ethanol are 120% for RGO–ZnO and 55% for ZnO. These results demonstrate that the presence of RGO results in a four times higher response to 100 ppm of acetone compared to pure ZnO. The RGO–ZnO response to the same concentration of ethanol is about 2.2 times higher compared to ZnO. The improvement of the sensing properties in the presence of RGO may be reasonably ascribed to the enhancement of the overall surface of the hybrid material, which would benefit the reactivity of both phases (ZnO and RGO) [33].

The hybrid material shows a higher response to acetone than to ethanol. From the formulas describing the sensing mechanism of acetone (Equations 2–4) and ethanol (Equation 2) and the sensing result of the RGO–ZnO towards the same concentrations of target gas seems that at 250 °C acetone releases more electrons than ethanol due to the interaction between the gas molecules and the adsorbed oxygen on the material surface. It may be one of the reasons of the better response to acetone. Besides, different gases have a different adsorption rate due the variation of adsorption energy.

**Figure 7:**
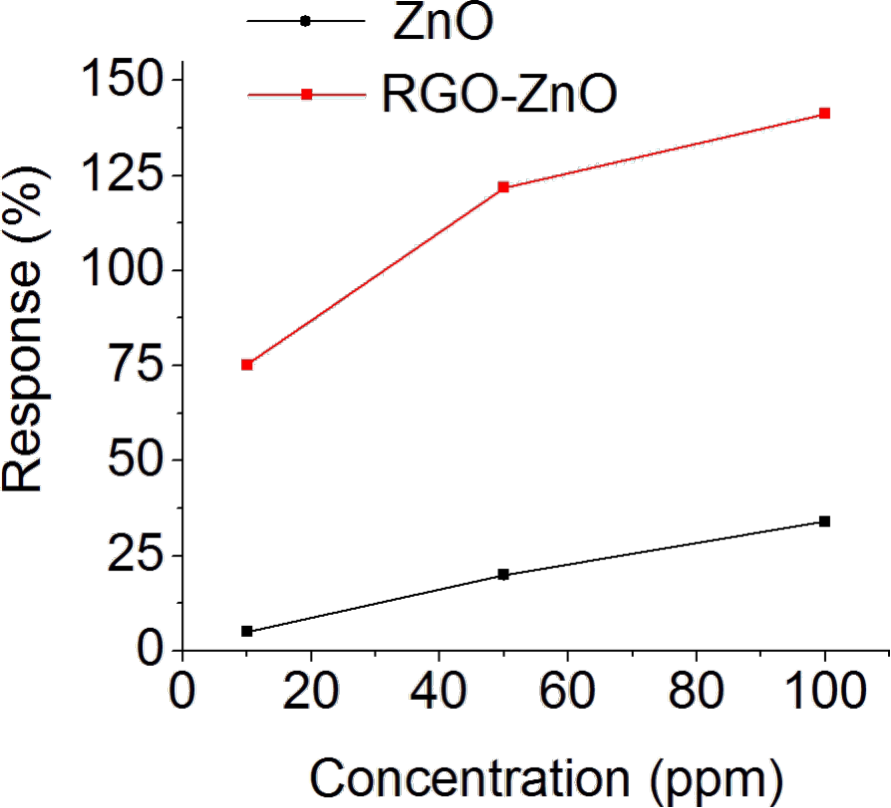
Calibration curve for acetone at an operating temperature of 250 °C and in a humid air background (RH = 50% @ 20 °C).

Figure 7 reports the calibration curves of the RGO–ZnO and pristine ZnO nanostructures for measuring acetone at a working temperature of 250 °C. The response for both structures shows good linearity with the concentration of acetone. The response of the hybrid structure towards all examined concentrations of acetone is greater compared to the pristine ZnO nanostructures. In addition to providing extra surface area for the adsorption sites and for the reaction with the analytes, the RGO platelets also may play a critical role in the electrical transport. RGO platelets reduce the height of the potential barrier for electron tunneling acting as a highly conductive electrical path for the transport of electrons through the nanostructure [34]. Therefore, RGO improves the sensing performance of ZnO. Due to this reason, in our future investigations we will study the sensing properties of the composite material varying the concentration and the reducing regimes of RGO in the structure to find the best regimes for the practical applications.

## Conclusion

In conclusion, chemiresistive gas sensors based on ZnO and RGO nanostructures with high sensing performance for the detection of VOCs have been developed. The sensing properties of the obtained structures have been investigated towards acetone and ethanol. To evaluate the sensing performance of the hybrid nanostructure we compared its properties with pristine ZnO nanostructures obtained with the same fabrication regimes. The sensing properties of ZnO have been improved because of the high surface area of RGO and nanostructured ZnO, as well as due to the ability of RGO to enhance the transport of charge carriers in the structure. Finally, incorporation of RGO into the metal oxide nanomaterials is a promising strategy in the detection of VOCs for environmental and health protection.

## References

[1] Ledenbach L H, Marshall R T, Sperber W H, Doyle M P (2009). Microbiological Spoilage of Dairy Products. Compendium of the Microbiological Spoilage of Foods and Beverages.

[2] Manolis A (1983). Clin Chem.

[3] Righettoni M, Tricoli A (2011). J Breath Res.

[4] Gossel T A, Bricker J D (1994). Principles of clinical toxicology.

[5] Righettoni M, Amann A, Pratsinis S E (2015). Mater Today.

[6] Di Natale C, Paolesse R, Martinelli E, Capuano R (2014). Anal Chim Acta.

[7] Galstyan V, Comini E, Ponzoni A, Sberveglieri V, Sberveglieri G (2016). Chemosensors.

[8] Comini E, Faglia G, Sberveglieri G (2009). Solid State Gas Sensing.

[9] Galstyan V, Comini E, Faglia G, Sberveglieri G (2013). Sensors.

[10] Devan R S, Patil R A, Lin J-H, Ma Y-R (2012). Adv Funct Mater.

[11] Morin F J (1959). Semiconductors.

[12] Kołodziejczak-Radzimska A, Jesionowski T (2014). Materials.

[13] Wang Z L (2004). J Phys: Condens Matter.

[14] Spencer M J S (2012). Prog Mater Sci.

[15] Kiriakidis G, Moschovis K, Kortidis I, Binas V (2012). Vacuum.

[16] Muhammad R, Rekha P, Mohanty P (2016). RSC Adv.

[17] Ionescu R, Espinosa E H, Leghrib R, Felten A, Pireaux J J, Erni R, Van Tendeloo G, Bittencourt C, Cañellas N, Llobet E (2008). Sens Actuators, B.

[18] Kholmanov I N, Domingues S H, Chou H, Wang X, Tan C, Kim J-Y, Li H, Piner R, Zarbin A J G, Ruoff R S (2013). ACS Nano.

[19] Schedin F, Geim A K, Morozov S V, Hill E W, Blake P, Katsnelson M I, Novoselov K S (2007). Nat Mater.

[20] Maarouf A A, Kasry A, Chandra B, Martyna G J (2016). Carbon.

[21] Dubal D P, Holze R, Gomez-Romero P (2014). Sci Rep.

[22] Galstyan V, Comini E, Kholmanov I, Faglia G, Sberveglieri G (2016). RSC Adv.

[23] Abideen Z U, Kim H W, Kim S S (2015). Chem Commun.

[24] Galstyan V, Comini E, Baratto C, Ponzoni A, Bontempi E, Brisotto M, Faglia G, Sberveglieri G (2013). CrystEngComm.

[25] Galstyan V, Comini E, Baratto C, Faglia G, Sberveglieri G (2015). Ceram Int.

[26] Stankovich S, Dikin D A, Dommett G H B, Kohlhaas K M, Zimney E J, Stach E A, Piner R D, Nguyen S T, Ruoff R S (2006). Nature.

[27] Ferrari A C (2007). Solid State Commun.

[28] Cancado L G, Pimenta M A, Saito R, Jorio A, Ladeira L O, Grueneis A, Souza A G, Dresselhaus G, Dresselhaus M S (2002). Phys Rev B.

[29] Yamazoe N, Shimanoe K (2008). Sens Actuators, B.

[30] Xu X L, Chen Y, Ma S Y, Li W Q, Mao Y Z (2015). Sens Actuators, B.

[31] Wang P, Wang D, Zhang M, Zhu Y, Xu Y, Ma X, Wang X (2016). Sens Actuators, B.

[32] Bagheri M, Khodadadi A A, Mahjoub A R, Mortazavi Y (2016). Sens Actuators, B.

[33] Russo P A, Donato N, Leonardi S G, Baek S, Conte D E, Neri G, Pinna N (2012). Angew Chem, Int Ed.

[34] Venkatesan A, Ramesha C K, Kannan E S (2016). J Phys D.

